# Prevention of post-operative delirium using an overnight infusion of dexmedetomidine in patients undergoing cardiac surgery: a pragmatic, randomized, double-blind, placebo-controlled trial

**DOI:** 10.1186/s13054-024-04842-1

**Published:** 2024-02-29

**Authors:** Olivier Huet, Thomas Gargadennec, Jean-Ferréol Oilleau, Bertrand Rozec, Nicolas Nesseler, Adrien Bouglé, Thomas Kerforne, Sigismond Lasocki, Vedat Eljezi, Géraldine Dessertaine, Julien Amour, Xavier Chapalain, the EXACTUM and the Atlanrea Study Group, Grégoire Le Gac, Nima Djavidi, Emmanuel Rineau, Dauphou Eddi, Emmanuel Novak, Maëlys Consigny, Karim Ashenoune, Antoine Roquilly, Philippe Seguin, Claire Dayot-Fitzellier, Francis Remerand, Marc Laffon

**Affiliations:** 1 Department of Anaesthesia, Intensive Care Medicine and Peri-Operative Medicine, Hôpital de la cavale Blanche, CHRU de Brest, Brest, France; 2 Department of Anaesthesia, Intensive Care Medicine and Peri-Operative Medicine, Hôpital Laennec, University Hospital Centre Nantes, Nantes, France; 3 Department of Anaesthesia, Intensive Care Medicine and Peri-Operative Medicine, University Hospital of Rennes, Rennes, France; 4 Department of Anaesthesia, Intensive Care Medicine and Peri-Operative Medicine, Institut de Cardiologie, Hôpital La Pitié-Salpêtrière, Paris, France; 5 Department of Anaesthesia, Intensive Care Medicine and Peri-Operative Medicine CHU de POITIERS, Poitiers, France; 6 Department of Anaesthesia, Intensive Care Medicine and Peri-Operative Medicine, CHU de ANGERS, I, Angers, France; 7 Department of Anaesthesia, Intensive Care Medicine and Peri-Operative Medicine, Hôpital Gabriel Montpied, CHU de Clermont Ferrand, Clermont Ferrand, France; 8 Department of Anaesthesia, Intensive Care Medicine and Peri-Operative Medicine, Grenoble Alpes University Hospital, Grenoble, France; 9 Department of Anaesthesia, Intensive Care Medicine and Peri-Operative Medicine, Hôpital Privé Jacques Cartier, Massy, France; 10 INSERM, Univ Rennes, CHU Rennes, Department of Anesthesia and Critical Care, CLCC Eugène Marquis, COSS [(Chemistry Oncogenesis Stress Signaling)] – UMR_S 1242, 35000 Rennes, France; 11 Sorbonne Université, GRC 29, Assistance Publique – Hôpitaux de Paris, DMU DREAM, Département d’Anesthésie Et Réanimation, Institut de Cardiologie, Hôpital La Pitié-Salpêtrière, Paris, France; 12 Service d’anesthésie-Réanimation Et Médecine Périopératoire CHU de ANGERS, Angers, France; 13 Direction de La Recherche Clinique Et de L’innovation, CHRU de Brest, Brest, France; 14 Department of Anaesthesia, Intensive Care Medicine and Peri-Operative Medicine, University Hospital Centre Nantes, Nantes, France; 15 Department of Anaesthesia, Intensive Care Medicine and Peri-Operative Medicine, CHU de POITIERS, Poitiers, France; 16 Department of Anaesthesia, Intensive Care Medicine and Peri-Operative Medicine, CHU de Tours, Tours, France

**Keywords:** Dexmedetomidine, Cardiac surgery, Delirium, Sleep quality

## Abstract

**Background:**

After cardiac surgery, post-operative delirium (PoD) is acknowledged to have a significant negative impact on patient outcome. To date, there is no valuable and specific treatment for PoD. Critically ill patients often suffer from poor sleep condition. There is an association between delirium and sleep quality after cardiac surgery. This study aimed to establish whether promoting sleep using an overnight infusion of dexmedetomidine reduces the incidence of delirium after cardiac surgery.

**Methods:**

Randomized, pragmatic, multicentre, double-blind, placebo controlled trial from January 2019 to July 2021. All adult patients aged 65 years or older requiring elective cardiac surgery were randomly assigned 1:1 either to the dexmedetomidine group or the placebo group on the day of surgery. Dexmedetomidine or matched placebo infusion was started the night after surgery from 8 pm to 8 am and administered every night while the patient remained in ICU, or for a maximum of 7 days. Primary outcome was the occurrence of postoperative delirium (PoD) within the 7 days after surgery.

**Results:**

A total of 348 patients provided informed consent, of whom 333 were randomized: 331 patients underwent surgery and were analysed (165 assigned to dexmedetomidine and 166 assigned to placebo). The incidence of PoD was not significantly different between the two groups (12.6% vs. 12.4%, *p* = 0.97). Patients treated with dexmedetomidine had significantly more hypotensive events (7.3% vs 0.6%; *p* < 0.01). At 3 months, functional outcomes (Short-form 36, Cognitive failure questionnaire, PCL-5) were comparable between the two groups.

**Conclusion:**

In patients recovering from an elective cardiac surgery, an overnight infusion of dexmedetomidine did not decrease postoperative delirium.

*Trial registration* This trial was registered on ClinicalTrials.gov (number: NCT03477344; date: 26th March 2018).

**Supplementary Information:**

The online version contains supplementary material available at 10.1186/s13054-024-04842-1.

## Background

During cardiac surgery, acute physiological changes induced by operative stress may lead to complications which increase ICU length of stay. Among these complications, post-operative delirium (PoD) is acknowledged to have a significant negative impact on patient outcome after cardiac surgery [1, 2]. PoD is characterized by an acute onset of mental status changes with fluctuating inattention, disorganized thinking and altered level of consciousness. There are growing evidence that delirium encompass multiple sub-phenotypes, consisting in a more complex syndrome that initially described [3, 4]. Among these sub-phenotypes, delirium occurring after cardiac surgery remains a crucial issue regarding its high incidence, with a rate of 12 to 55% [5, 6]. After an elective cardiac surgery, patients exhibiting PoD are at greater risk of death, readmission to the hospital, cognitive and functional decline, and a lower quality of life after hospital discharge [7]. Therefore diagnosis, treatment and prevention of PoD after cardiac surgery are the subject of intensive clinical research [8].

To date there is no specific treatment for PoD [9]. Prevention of PoD mainly relies on patient re-orientation, pain control and preservation of nictemeral rhythm [10]. Following cardiac surgery or cardiopulmonary bypass, several risk factors associated to the occurrence of PoD have been reported [2, 11]. Among modifiable risk factors, perioperative sleep disturbances have been closely associated with PoD [12].

Dexmedetomidine is an α-2A adrenergic receptor agonist often used in anaesthesia and critical care medicine to sedate patients. In comparison with GABA-activating drugs, such as benzodiazepines, dexmedetomidine preserves better normal sleep architecture, as it produces spindle and slow-delta oscillations patterns close to N2 sleep stage [13]. According to a recent systematic review, dexmedetomidine is the most frequently studied pharmacological agent to prevent PoD after cardiac surgery [8]. Turan et al. have tested the prophylactic effect of dexmedetomidine on PoD and supra-ventricular rhythm abnormalities after cardiac surgery without showing a beneficial effect on PoD prevention [14]. More recently, Qu et al. reported that a single overnight administration of dexmetedomidine decreased the incidence of delirium at day one after cardiac surgery [15]. The conflicting results of these trials could be explained by a significant heterogeneity between the studies designs and a lack of consistency in PoD definition precluding to draw a definitive conclusion on the benefit to risk ratio of the use of dexmedetomidine infusion in this context. Moreover, the significant increase of adverse events reported during dexmedetomidine infusion needed to be confirmed [14, 16].

We have designed a randomized double-blind placebo-controlled trial to determine whether a repeated nocturnal infusion of a low dose of dexmedetomidine prevents the onset of PoD in patients after an elective cardiac surgery.

## Methods

### Trial design and setting

We conducted a pragmatic, randomized, double-blind, parallel group, placebo-controlled trial. Nine centres in France participated to the study. The study protocol and statistical analysis plan have been extensively described and previously published [20].

Screening for eligibility was performed prior to a planned consultation with an anaesthesiologist a few weeks before surgery. All eligible patients were asked for consent and included in the study. The inclusion criteria were patient aged 65 years or older who underwent cardiac surgery with or without cardiopulmonary bypass. Patients were excluded if they met the following criteria: documented cognitive failure or dementia, patients previously included in a study on sedation or analgesia, predicted length of stay in the ICU < 24 h, alpha-2 agonists allergy or intolerance, emergency surgery for immediate life threatening situation, uncontrolled hypotension, 2nd or 3rd grade atrio ventricular block in the absence of a pacemaker, hepatocellular insufficiency defined by the presence of the diagnosis in medical records, altered hepatic tests defined by abnormal values of laboratory test, acute cerebrovascular disease, patients receiving Clonidine, patients under guardianship or curatorship.

Randomisation was centralized and performed by the independent clinical research unit at the Brest University Hospital. A blocked randomisation with varying block sizes was performed. Randomization was also stratified on centres and planned modality of surgery between ‘on pump’ and ‘off pump’. Before surgery, patients were randomly assigned (1:1), by local investigators, either in the dexmedetomidine group or the placebo group using a dedicated and protected website (CSOnline; Clinsight). All the randomisation process allows a rapid and concealed treatment assignment from patients and site investigators.

### Intervention

The study drugs, dexmedetomidine (100 µg/ml) and placebo (sodium chloride 0.9%), were conditioned in vials of 2 ml. The size and shape of the two vials, the colour and texture of the two treatments are strictly identical. Blinding was performed by the pharmacist at the coordinating centre by erasing the original label of the vials and then labelling them accordingly to study treatment.

For all participants, a continuous infusion of dexmedetomidine or matching placebo was started the day of surgery from 8 pm to 8 am on the next day. Minimal infusion rate was 0.1 µg/kg/h and maximum 1.4 µg/kg/h. Infusion rate was modified by the treating nurse or the clinician by 0.1 µg/kg/h every hours with an objective of a Richmond Agitation and Sedation Scale (RASS) from − 1 to + 1. From the day of surgery, the treatment was administered every night until the patient was discharge from ICU or stopped after 7 days if the patient remained in ICU. Open label use of dexmedetomidine were not authorized. Administration of clonidine was not allowed in both groups.

Other medical interventions, especially sedatives/analgesics were left at the discretion of clinicians. The nine participating centres are high-volume cardiac surgery centres. Local investigators follow the latest French guidelines on enhanced recovery after cardiac surgery endorsed by the French Society of Anaesthesia and Intensive Care medicine and the French Society of Thoracic and Cardiovascular Surgery [17]:

- All centres apply a minimally invasive extracorporeal circulation approach to reduce postoperative complication
- The type cardioplegia was not protocolized
- Intravenous propofol or halogenated inhaled anaesthesia was used intraoperatively
- A protective ventilation was applied with a tidal volume ranged from 6 to 8 ml/kg.
- Multimodal analgesia, using locoregional technique and co-analgesics was used
- After surgery, patients were admitted under mechanical ventilation in all participating ICU and an enhanced recovery program was followed: earliest possible extubation (< 6 h), early mobilisation, removing chest drain and catheter as soon as possible.

In all participating centre, delirium prevention relied on ABCDE bundle.

### Primary and secondary outcomes

The primary outcome was the occurrence of PoD, evaluated by the CAM-ICU, within the 7 days after surgery. Before the beginning of the study, staffs and research personnels were trained to performed CAM-ICU with the French version of the CAM-ICU Training Manual [21]. During the study, the CAM-ICU was measured twice daily during the 7 days following surgery. Measurements were performed in the morning between 8 and 12 am and in the afternoon between 4 and 8 pm. At the time of evaluation, dexmedetomidine or placebo was no longer administered since it was strictly a night infusion. If patients were discharged from ICU before the 7th day after surgery, twice daily PoD evaluation was performed in the surgical ward with the same modalities. A PoD event was considered if at least one of all the CAM-ICU evaluations was positive within 7 days after surgery.

Secondary efficacy outcomes included agitation-sedation status evaluated by the Richmond Agitation-Sedation Scale (RASS) until day 7, occurrence of agitation-related adverse events until day 7 (defined by an unplanned extubation, a medical device removal, a falling out of bed, an ICU runaway, an immobilisation device removal, a self-aggression and/or an agrgession towards medical staff), sleep quality evaluated with a numerical scale (from 0 to 10) until day 7, quality of sleep with the Leeds Sleep Evaluation Questionnaire (LSEQ) until day 7, ICU length of stay, hospital length of stay and hospital mortality. Long-term functional outcomes were also evaluated at 3 months: quality of life with Short-Form 36 questionnaire (SF-36) [18, 19], cognitive function with the Cognitive Failure Questionnaire (CFQ), occurrence of post-traumatic stress disorder (PTSD) evaluated by the Post Traumatic Stress Disorder Checklist (PCL-5) questionnaire (CFQ, PCL-5 and LSEQ are described in Additional file 1: Appendix 1 to 3). As preplanned ancillary analysis, we also evaluated the effect of dexmedetomidine on supra ventricular arrhythmias [20]. Due to the relevance of this outcome, it was finally decided to include this ancillary analysis in the article.

Secondary safety outcomes included bradycardia, hypotension, arrythmia and renal failure (assessed by the renal sub-part of the SOFA score) within the 7 days after surgery.

### Sample size calculation

According to recent clinical trials, we hypothesized a PoD incidence of 25% in the studied population [2, 14]. We expected a 50% decrease in incidence of delirium in the dexmedetomidine group. Data from previous clinical trials showed that such treatment effect is clinically relevant [20–22]. We calculated that 332 patients would be needed to detect this difference with a 5% one-tailed type I error and a power of 90%. Considering a lost to follow-up rate of 5%, the final sample size was fixed at 348 patients. Sample size calculation was performed using SAS statistical software (version 9.4).

### Statistical analysis

For continuous variables, baseline characteristics of patients were described with mean and standard deviation for normally distributed variables, or median and interquartile range for the other continuous variables. Number and frequencies were used for categorical variables description.

Primary outcome analysis was performed with Chi-square test in the intention-to-treat population. No interim analysis was performed. If the occurrence of PoD is missing for a patient, we have considered that the patient had no PoD within 7 days. We performed a sensitivity analysis of the primary outcome without missing values for PoD. A post hoc analysis was also performed to evaluate the number of number of days alive without delirium at Day 7 in both groups in the overall population and also in the sub-group of patients with delirium. For this analysis, the number of number of days alive without delirium was defined as the number of days alive without any positive CAM-ICU at Day 7. If a patient had both a positive and a negative CAM-ICU in the same day, we considered this case as a positive for PoD at that day. This post-hoc analysis was performed with a Wilcoxon test.

Secondary outcomes analysis was also performed in the intention-to-treat population, with Student t test for continuous variables and Chi-square test for categorical variables.

For the primary outcome, analysis was also performed in the following subgroups: modality of surgery (by-pass vs. off-pump) and type of cardiac sugery (valvular, coronary or combined surgery).

As an exploratory analysis, we also evaluated PoD and sleep quality (LSEQ and numerical scale), in the as-treated population (i.e., patients who received dexmedetomidine or placebo the night before the evaluation).

Statistical analysis was performed using SAS statistical software (version 9.4), except for figures which were made with R statistical software (version 3.6.1).

## Results

**Fig. 1:**
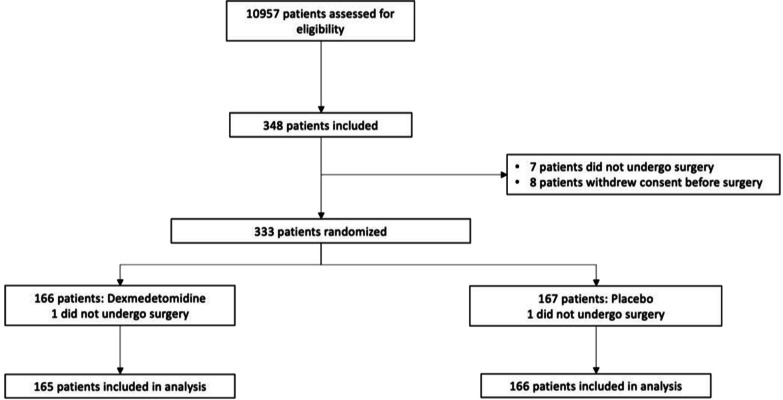
Flowchart of the study

Patients were enrolled from January 2019 to July 2021. Among the 10 957 patients screened a total of 348 patients provided informed consent and were included into the study: 7 patients did not undergo surgery and 8 patients withdrew consent before surgery. A total of 333 patients were randomized: 167 in the placebo group and 166 in the dexmedetomidine group. Two patients were randomized (one in each group) but did not undergo surgery. A total of 331 patients underwent surgery and were subsequently analysed as the intention-to-treat population. The flowchart of the study is represented in Fig. 1. Baseline characteristics of the patients are summarized in Table 1. The median duration of dexmedetomidine (or placebo) infusion was 3 days [2–4] in both groups. Median and mean infusion rate of dexmedetomidine (or placebo) were reported in Additional file 1: Table S1 and were similar between both groups. Main analgesic, sedative and psychotropic medications administered at least once to the patients are reported in Additional file 1: Table S2.

**Table 1:** Baseline characteristics in the intention-to-treat population

Characteristics	Total (*n* = 331)	Dexmedetomidine group (*n* = 165)	Placebo group (*n* = 166)
Preoperative characteristics			
Age (year), mean (SD)	73 (5)	73 (5)	73 (5)
Female sex, *n* (%)	80 (24.2)	37 (22.4)	43 (25.9)
Body weight (kg), mean (SD)	78 (15)	79 (17)	76 (13)
Euroscore, mean (SD)	4.3 (2.8)	4.3 (2.7)	4.2 (2.8)
Pre-Deliric score, mean (SD)	16.4 (7.6)	16.8 (7.9)	16 (7.3)
Coronary artery disease, *n* (%)	181 (55.2)	89 (54.9)	92 (55.4)
Chronic heart failure, *n* (%)	61 (18.6)	29 (17.9)	32 (19.3)
Left ventricular ejection fraction, mean (SD)	60 (9.8)	60.5 (10.1)	59.4 (9.5)
Treated hypertension, *n* (%)	232 (70.7)	118 (72.8)	114 (68.7)
Arrhythmia^a^, *n* (%)	63 (19.2)	31 (19.1)	32 (19.3)
Pulmonary hypertension, *n* (%)	29 (9.1)	15 (9.4)	14 (8.9)
Diabetes, *n* (%)	74 (22.4)	30 (18.2)	44 (26.5)
Renal function, *n* (%)			
Normal	285 (86.1)	139 (84.2)	146 (88)
Mildly impaired (GFR: 60–89 ml/min)	14 (4.2)	10 (6)	4 (2.4)
Moderately impaired (GFR: 30–59 ml/min)	26 (7.9)	12 (7.3)	14 (8.4)
Severely impaired (GFR < 30 ml/min)	3 (0.9)	2 (1.2)	1 (0.6)
Respiratory disease, *n* (%)	78 (23.6)	46 (27.9)	32 (19.3)
Obstructive sleep apnoea	39 (11.8)	22 (13.3)	17 (10.2)
COPD	25 (7.6)	16 (9.7)	9 (5.4)
Asthma	17 (5.1)	9 (5.5)	8 (4.8)
Stroke, *n* (%)	35 (10.7)	20 (12.3)	15 (9)
Operative characteristics			
Type of surgery, *n* (%)			
CABG	181 (54.7)	93 (56.4)	88 (53)
Aortic valve replacement	140 (42.3)	62 (37.6)	78 (47)
Mitral valve replacement	38 (11.5)	20 (12.1)	18 (10.8)
Aortic surgery	26 (7.9)	13 (7.9)	13 (7.8)
Combined surgery (CABG and valve)	53 (16)	25 (15.2)	28 (16.9)
Cardiopulmonary bypass, *n* (%)	314 (95.4)	155 (94.5)	159 (95.8)
Bypass duration (min), mean (SD)	110 (55)	113 (55)	108 (55)
Blood transfusion, *n* (%)			
RBC	51 (19.7)	25 (19.4)	26 (20)
FFP	23 (8.9)	13 (10.1)	10 (7.7)
Vasoactive drugs, *n* (%)			
Norepinephrine	246 (95)	122 (94.6)	124 (95.4)
Epinephrine	9 (3.5)	5 (3.8)	4 (3.1)
Dobutamine	47 (18.1)	24 (18.5)	23 (17.7)

Data are expressed as mean (standard deviation) or number (percentage) as appropriate

*CABG* Coronary Artery Bypass Graft,* COPD* Chronic Obstructive Pulmonary Disease,* GFR* Glomerular Filtration Rate,* FFP* Fresh Frozen Plasma,* Pre-Deliric* PREdiction of DELIRium in ICu patients,* RBC* Red Blood Cell,* SD* Standard Deviation

^a^This comorbidity encompass atrial fibrillation and atrial flutter

### Primary outcome

In the intention-to-treat population, PoD occurred in 40 patients (12.5%): 20 of 165 patients assigned to the dexmedetomidine group (12.6%) and 20 of 166 patients assigned to the placebo group (12.4%), *p* = 0.97 (Table 2). Eleven PoD assessments were missing: 6 patients in dexmedetomidine and 5 in the placebo group had no CAM-ICU evaluation within 7 days. Main reason why CAM-ICU assessment was missing from Day 1 to Day 7 are reported in the Additional file 1: Table S3. When considering missing values for PoD without imputation, sensitivity analysis did not find any difference between the two groups: 14 (8.5%) versus 15 (9%), *p* = 0.97. In the as-treated population (*n* = 312), PoD occurred in 27 patients (8.9%): 13 of 153 patients assigned to the dexmedetomidine group (8.7%) and 14 of 159 patients assigned to the placebo group (9.1%), *p* = 0.9 (Table 2). In the post hoc analysis, the median number of delirium free days at Day 7 were similar in both groups: 7 [7] versus 7 [7], *p* = 0.98. In the sub-group of patients with delirium (*n* = 40), there was also no difference in the median number of delirium free-days: 6 [5, 6] versus 6 [4.5–6], *p* = 0.44.

**Table 2:** Outcomes in the study participants assigned to dexmedetomidine or placebo group. Data are expressed as number (%), mean (SD) or median (IQR) as appropriate

Outcomes	Total (*n* = 331)	Dexmedetomidine group (*n* = 165)	Placebo group (*n* = 166)	*P*
**Primary outcome**				
PoD within the 7 days after surgery, *n* (%)	40 (12.5)	20 (12.6)	20 (12.4)	0.97
Missing	11	6	5	
**Secondary outcomes**				
RASS^a^, mean (SD)				
Minimum RASS score	− 0.5 (0.8)	− 0.6 (0.9)	− 0.5 (0.7)	0.76
Maximum RASS score	− 0.1 (0.7)	− 0.2 (0.8)	− 0.1 (0.6)	0.92
Missing	10	6	4	
Agitation-related adverse events, n (%)	29 (8.7)	15 (9.1)	14 (8.4)	0.83
Sleep quality evaluation				
Numerical scale, median (IQR)				
Intention-to-treat population	5.3 (4.3–6.4)	5.2 (4.3–6.4)	5.3 (4.3–6.4)	0.98
Missing	32	13	19	
As-treated population^b^	5.3 (3.5–7.0)	5.7 (4.0–7.0)	5.0 (3.0–6.8)	0.01
Missing	29	11	18	
LSEQ, median (IQR)				
Intention-to-treat population	− 4.0 (− 9.3–3.5)	− 4.0 (− 8.9–3.7)	− 4.2 (− 9.9–3.2)	0.32
Missing	27	13	14	
As-treated population^b^	− 5.0 (− 11.8–3.0)	− 4.0 (− 10.6–4.0)	− 7.3 (− 14.8–1.5)	0.02
Missing	30	14	16	
Functional outcomes evaluated at 3 months				
Short-Form 36 ^c^, mean (SD)				
Physical component	44.1 (8.5)	45 (8.9)	43.3 (8.1)	0.13
Mental component	50.1 (9.8)	51 (9.5)	49.3 (10.1)	0.23
Missing	92	46	46	
Cognitive Failure Questionnaire^d^, mean (SD)	24.5 (13.2)	23.7 (13.6)	25.4 (12.9)	0.45
Missing	111	51	60	
PTSD, *n* (%)	8 (3.6)	2 (1.9)	6 (5.2)	0.28
Missing	109	59	50	
**Other outcomes**				
ICU length of stay, median (IQR)	3 (2–5)	3 (2–5)	3 (2–5)	0.85
Hospital length of stay, median (IQR)	11 (8–15)	11 (8–16)	11 (8–15)	0.83
Hospital mortality, *n* (%)	6 (1.8)	5 (3)	1 (0.6)	0.12
Mortality at 3 months, *n* (%)	11 (3.3)	8 (4.8)	3 (1.8)	0.12

*CAM-ICU* Confusion Assessment Method for the Intensive Care Unit, *LSEQ* Leeds Sleep Evaluation Questionnaire, *PoD* Postoperative Delirium, *PTSD* Post Traumatic Stress Disorder, *RASS* Richmond Agitation-Sedation Scale, *SD* Standard Deviation

^a^Mean of the minimum and maximum RASS score was summarized in this table

^b^As-treated analysis (*n* = 312) was done on patients who received the treatment, dexmedetomidine (*n* = 153) or placebo (*n* = 159), the night before the sleep quality evaluation

^c^For SF-36 questionnaire: a higher mean score in physical and mental components are related to higher quality of life

^d^For cognitive failure questionnaire: a higher mean score is related to more cognitive dysfunction (see appendix 1 for more details)

### Secondary outcomes

Minimum and maximum RASS score were comparable between the two groups (Table 2). The incidence of agitation-related adverse events was non-significant between the two groups: 15 of 165 patients assigned to the dexmedetomidine (9.1%) and 14 of 166 patients assigned to the placebo group (8.4%), *p* = 0.83 (Table 2). In the intention-to-treat population, there were no difference in sleep quality evaluation neither on numerical scale (median score: 5.2 vs. 5.3, *p* = 0.98) nor on LSEQ (median score: − 4 vs. − 4.2, *p* = 0.32) (Table 2). Differences in daily evaluation of the LSEQ are shown in Additional file 1: Table S4. Details of the LSEQ different sections during the 7 days of observation are shown in Additional file 1: Table S5. Main reasons why sleep quality evaluation was not performed or missing are shown in Additional file 1: Table S3.

There was no significant effect of dexmedetomidine on ICU length of stay, hospital length of stay and 90 days mortality (Table 2). There was no difference between the two groups regarding long-term postoperative functional outcomes (Short-Form 36, cognitive failure and incidence of PTSD). The incidence of supra-ventricular arrhythmias was comparable between the two groups: 74 of 165 patients assigned to the dexmedetomidine (44.8%) and 65 of 166 patients assigned to the placebo group (39.2%), *p* = 0.29.

**Fig. 2:**
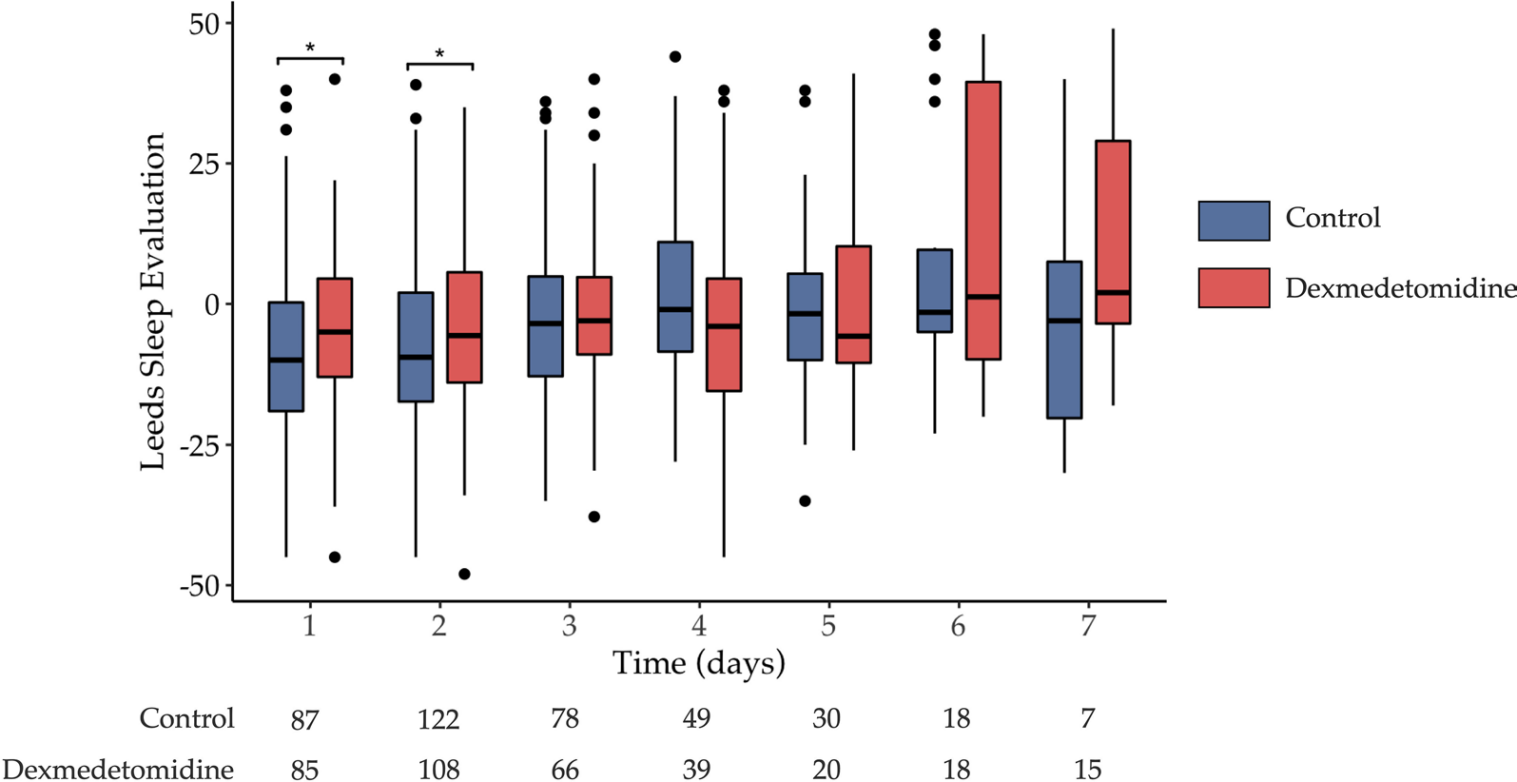
Daily evaluation of sleep quality by the LSEQ (Leeds Sleep Evaluation Questionnaire) in as-treated population. The horizontal lines in the centre of each boxes indicates the median; lower and upper hinges correspond respectively to the first and third quartiles (the 25th and 75th percentiles); upper whisker extends from the hinge to the largest value no further than 1.5 times IQR from the hinge (where IQR is the inter-quartile range, or distance between the first and third quartiles); lower whisker extends from the hinge to the smallest value at most 1.5 times IQR of the hinge. Data beyond the end of the whiskers represents outliers and are plotted individually. *: *p* < 0.05, results are expressed with box and whiskers

In the as-treated population, sleep quality evaluated by numerical scale was significantly improved in the patients receiving dexmedetomidine (5.7 vs 5; *p* = 0.01) (Table 2). Moreover, the average scores of LSEQ were also significantly improved for patients receiving dexmedetomidine compared to those receiving placebo (− 4.0 vs. − 7.3; *p* = 0.02) (Table 2). Evolution of the LSEQ in the as-treated population is shown in Fig. 2.

### Adverse events

Patients receiving dexmedetomidine had significantly more episode of hypotension compared to the patients in the placebo group (7.3% vs. 0.6%; *P* < 0.01). The occurrence of bradycardia was higher in the dexmedetomidine group compared to placebo, but the difference did not reach significancy (3.6% vs 0.6%; *P* = 0.07). These results are summarized in Table 3. Finally, no additional renal failure assessed by the renal component of the SOFA score were associated with the use of dexmedetomidine compared to placebo. The daily assessment of renal component of the SOFA score was displayed in the Additional file 1: Table S6.

**Table 3:** Safety outcomes and arrythmia within the 7 days in patients assigned to dexmedetomidine and placebo group

	Total (*n* = 331)	Dexmedetomidine group (*n* = 165)	Placebo group (*n* = 166)	*P*
Hypotension				
Yes	13 (3.9)	12 (7.3)	1 (0.6)	< 0.01
No	318 (96.1)	153 (92.7)	165 (99.4)	
Bradycardia				
Yes	7 (2.1)	6 (3.6)	1 (0.6)	0.07
No	324 (97.9)	159 (96.4)	165 (99.4)	
Ventricular arrythmia				
Yes	21 (6.3)	8 (4.8)	13 (7.8)	0.27
No	310 (93.7	157 (95.2	153 (92.2)	
Supra ventricular arrythmia				
Yes	139 (42)	74 (44.8)	65 (39.2)	0.29
No	192 (58)	91 (55.2)	101 (60.8)	

Data are expressed as number (percentage)

### Subgroup analysis

There was no difference in PoD in the five preplanned sub-group analysis. Incidence of PoD was similar whenever the modality of surgery or type of cardiac surgery were. The results of subgroup analysis are shown in Additional file 1: Table S7.

## Discussion

We report that an overnight infusion of dexmedetomidine did not prevent the onset of postoperative delirium after elective cardiac surgery. In our study, dexmedetomidine did not improve the long-term functional outcome nor the incidence of acute heart arrhythmias, but more episodes of hypotension were reported in the dexmedetomidine-treated patients.

Dexmedetomidine is central highly selective short-acting alpha-2 adrenoreceptor agonist with anxiolytic, sympatholytic and sedative properties. Recent studies have shown a potential beneficial effect of dexmedetomidine in mitigating surgical stress, by acting as co-analgesic [23, 24], reducing inflammation state, improving immune function [25] and restoring sleep architecture in post-operative period [13]. All these theoretical beneficial effects may improve perioperative care for cardiac surgery patients through ERAS (Enhanced Recovery After Surgery) programs [24].

Among all beneficial effect of dexmedetomidine, prevention of PoD after cardiac surgery has previously been described by several interventional studies [26]. These results seem strengthen by recent meta-analysis reporting a positive effect of dexmedetomidine infusion to prevent PoD after cardiac surgery [27, 28]. However, a critical appraisal of the existing literature has pointed out the relatively small sample for the considered studies, the single centre design for a majority of them and the high heterogeneity in their findings [8, 26]. Finally, the protocols for dexmedetomidine administration varied greatly from one study to another, which preclude any valuable conclusion of the actual administration protocol [27]. Especially, the duration of the treatment also differed but never lasted more than 24 h. Moreover, dexmedetomidine infusion was also combined to other sedative or analgesic drugs in several studies. Recently, two randomized controlled trial have tested the prophylactic effect of dexmedetomidine infusion in a peri-operative setting and showed contradictory results [14, 15].

In the DECADE study, Turan et al. suggested that the anti-inflammatory properties of a low dose of dexmedetomidine may decrease PoD onset [14]. In this study, the drug was infused before the start of cardiac surgery and continued for 24 h [14]. However, the infusion of dexmedetomidine did not decrease delirium onset in the treated group [14]. We believe that the results of our study and the DECADE study are complementary as we tested a similar population but postulated a different mechanism of action [14]. In addition, we confirmed the results the DECADE study about the absence of effect of dexmedetomidine on the occurrence of arrythmia after cardiac surgery [14]. Our main findings are also in line with the results of a recent meta-analysis which failed to demonstrate any beneficial effect of dexmedetomidine to prevent PoD [29]. In the MINDSS study, Qu et al. reported that a single bolus of dexmedetomidine administered at nighttime could prevent delirium by promoting sleep [15]. However, no difference was found regarding sleep quality. As dexmedetomidine is a short acuting drug (half-life: 2 h), a single bolus may not be sufficient to promote a better sleep quality. Therefore the positive result reported by the authors may not be explained by the sedative effect of the drug [15].

To date, few clinical trials have evaluated long term functional outcomes, especially cognitive functions, in patients treated with dexmedetomidine after cardiac surgery [14, 15]. Association between delirium and impaired long-term outcome has been reported, as dexmedetomidine may prevent PoD it may also be associated with an improvement in cognitive function [30]. In the MINDSS trial, the authors reported a difference in terms of PoD, but they did not demonstrate any difference in terms of cognitive function at day 90 and day 180. In our study, we did not find any effect of dexmedetomidine on quality of life and cognitive failure 3 months after surgery. It also has been reported that dexmedetomidine could exert a protective effect against fear memory and anxiety behaviour, potentially preventing PTSD [31–33]. In our study, the proportion of PTSD, as an exploratory outcome, was less important in the patients receiving dexmedetomidine compared to placebo (1.9% vs. 5.2%), but the result was not statistically significant. This exploratory result may be of interest for future studies in the context of cardiac surgery.

Dexmedetomidine is known to increase the incidence of hypotensive events in cardiac surgery patients [14]. Our study is the third randomized trial reporting a significant increase of hypotensive events in patients receiving a prophylactic infusion of dexmedetomidine to attempt to prevent PoD. Therefore, the use of dexmedetomidine in this context should carefully be considered.

### Strengths and limitations of the study

Randomized, double-blind, placebo-controlled design remains the main strength of our study. Regarding the number of participating centres, our findings are also generalizable to the other units. Moreover, patients’ characteristics are similar to those include in a recent large multicentre French cohort [34]. As our study was conducted as a pragmatic trial, our findings are also largely applicable in cardiac surgery setting. Finally, our study is the first one which documented a potential effect of dexmedetomidine on long-term cognitive function in cardiac surgery setting.

Our study has also some limitations. First, the incidence of PoD was lower than expected so our study suffers from lack of power. We observed an incidence that is comparable to the incidence found recently in the DECADE trial (12% vs. 17%) [14], but it was higher than the PoD incidence reported in the MINDSS trial (8.5% vs. 2.9%) [15]. These discrepancies between RCTs reinforce the need for further open-access, large, integrated and scalable registries in cardiac surgery to inform trialists of outcome incidences. The effect of the intervention observed in our study is close to nil (between-group difference: 0.2%) and far less important than the effect size chosen for the study sample size calculation. So, our study was underpowered to identify such small effect size. The evaluation of sleep quality was not performed using polysomnography, which is the gold standard technology to assess sleep quality and diagnose sleep disturbance. However, this method cannot be applied to a large number of patients in a typical ICU environment. The LSEQ is a self-rating questionnaire that has been widely used and is considered to be robust and reliable and can be used as a surrogate for polysomnography [35, 36]. Our data show consistency between the results using LSEQ and the sleep quality numerical scale. Sample size calculation was performed using one-sided alpha (5%). It ignores the possibility that dexmedetomidine may increase PoD or increase ICU stay by over-sedating the patients. To our knowledge, such side effects have not been reported in the literature. A two-tailed alpha remains the statistical gold standard, but it this particular case it would not have influence the result of our study. Finally, approximatively one third of the patients received at least once some benzodiazepine during their ICU stay, although this is balanced between the two groups it could have mitigate the effect of the intervention.

## Conclusion

In this multicentre, randomized, double-blind, controlled trial, prophylactic overnight infusion of dexmedetomidine failed to decrease the incidence of PoD after cardiac surgery. It had no effect on long-term functional outcomes. On the other hand, it significantly increased the risk of hypotension. Thus, dexmedetomidine should not be given to prevent PoD after an elective cardiac surgery.

### Supplementary Information

**Additional file 1. Table S1**. Minimum and maximum dose of dexmedetomidine and its corresponding placebo administered every night (from 8 pm to 8 am) from Day 0 to Day 7.** Table S2**. All concomitant treatments administered from inclusion to Day 7 in both groups.** Table S3**. Main reason why CAM-ICU assessment and sleep quality evaluation were not performed or missing from Day 1 to Day 7.** Table S4**. Comparison of dexmedetomidine versus placebo on secondary outcomes: daily evaluation of sleep quality. Data are expressed as median and IQR.** Table S5**. Comparison of dexmedetomidine versus placebo on secondary outcomes: detailed sections of LSEQ during the 7 days of observation. Data are expressed as median and IQR.** Table S6**. Baseline creatinine level and daily renal component of the SOFA (Sequential Organ Failure Assessment) score. **Table S7**. Preplanned sub-group analysis for the primary outcome. Occurrence of PoD within the 7 days after surgery are expressed as number (%).** Appendix 1**. The cognitive failures questionnaire.** Appendix 2**. The PCL-5 standard form checklist.** Appendix 3**. The Leeds Sleep Evaluation Questionnaire (LSEQ). Each item is rated from -5 to +5. Negative score corresponded to negative effects on sleep quality.

## Data Availability

Individual, deidentified participant data, including data dictionaries, may be shared. Templates of the informed consent forms may be shared upon request. The data will be available following publication, with no end date, and will be shared with anyone who wishes to access them with a clear data sharing agreement, for any purpose of analyses. For data access, please contact the corresponding author.

## Abbreviations

CABG: Coronary Artery Bypass Graft
CAM-ICU: Confusion Assessment Method for the Intensive Care Unit
ICU: Intensive Care Unit
LSEQ: Leeds Sleep Evaluation Questionnaire
PCL-5: Post Traumatic Stress Disorder Checklist
PoD: Postoperative Delirium
PRE-DELIRIC: PREdiction of DELIRium in ICu patients
PTSD: Post Traumatic Stress Disorder
RASS: Richmond Agitation-Sedation Scale
SD: Standard deviation
SF-36: Quality of life Short Form 36
