# Supplementary material for: Prevention of post-operative delirium using an overnight infusion of dexmedetomidine in patients undergoing cardiac surgery: a pragmatic, randomized, double-blind, placebo-controlled trial
Source: Crit Care. 2024 Feb 29;28:64. doi: 10.1186/s13054-024-04842-1 (PMC10902989; doi:10.1186/s13054-024-04842-1)
Supplement: Supplementary file 1 — Additional file 1. Table S1. Minimum and maximum dose of dexmedetomidine and its corresponding placebo administered every night (from 8 pm to 8 am) from Day 0 to Day 7. Table S2. All concomitant treatments administered from inclusion to Day 7 in both groups. Table S3. Main reason why CAM-ICU assessment and sleep quality evaluation were not performed or missing from Day 1 to Day 7. Table S4. Comparison of dexmedetomidine versus placebo on secondary outcomes: daily evaluation of sleep quality. Data are expressed as median and IQR. Table S5. Comparison of dexmedetomidine versus placebo on secondary outcomes: detailed sections of LSEQ during the 7 days of observation. Data are expressed as median and IQR. Table S6. Baseline creatinine level and daily renal component of the SOFA (Sequential Organ Failure Assessment) score. Table S7. Preplanned sub-group analysis for the primary outcome. Occurrence of PoD within the 7 days after surgery are expressed as number (%). Appendix 1. The cognitive failures questionnaire. Appendix 2. The PCL-5 standard form checklist. Appendix 3. The Leeds Sleep Evaluation Questionnaire (LSEQ). Each item is rated from -5 to +5. Negative score corresponded to negative effects on sleep quality. [file 13054_2024_4842_MOESM1_ESM.docx]

**Supplementary material**

**Table S1:** Minimum and maximum dose of dexmedetomidine and its corresponding placebo administered every night (from 8 pm to 8 am) from Day 0 to Day 7.

|  | **Dexmedetomidine group** | **Placebo group** |
| --- | --- | --- |
|  | **(n=165)** | **(n=166)** |
| **From Day 0 to Day 1** |  |  |
| Min |  |  |
| Median [IQR] | 0.10 [0.10, 0.10] | 0.10 [0.10, 0.10] |
| Mean (SD) | 0.11 (0.03) | 0.12 (0.06) |
| Max |  |  |
| Median [IQR] | 0.10 [0.10, 0.10] | 0.10 [0.10, 0.10] |
| Mean (SD) | 0.16 (0.14) | 0.13 (0.07) |
| **From Day 1 to Day 2** |  |  |
| Min |  |  |
| Median [IQR] | 0.10 [0.10, 0.10] | 0.10 [0.10, 0.10] |
| Mean (SD) | 0.12 (0.04) | 0.12 (0.05) |
| Max |  |  |
| Median [IQR] | 0.10 [0.10, 0.20] | 0.10 [0.10, 0.23] |
| Mean (SD) | 0.23 (0.22) | 0.18 (0.14) |
| **From Day 2 to Day 3** |  |  |
| Min |  |  |
| Median [IQR] | 0.10 [0.10, 0.10] | 0.10 [0.10, 0.10] |
| Mean (SD) | 0.12 (0.04) | 0.12 (0.05) |
| Max |  |  |
| Median [IQR] | 0.10 [0.10, 0.19] | 0.10 [0.10, 0.20] |
| Mean (SD) | 0.20 (0.15) | 0.18 (0.13) |
| **From Day 3 to Day 4** |  |  |
| Min |  |  |
| Median [IQR] | 0.10 [0.10, 0.10] | 0.10 [0.10, 0.10] |
| Mean (SD) | 0.11 (0.03) | 0.12 (0.04) |
| Max |  |  |
| Median [IQR] | 0.10 [0.10, 0.20] | 0.10 [0.10, 0.20] |
| Mean (SD) | 0.22 (0.23) | 0.16 (0.11) |
| **From Day 4 to Day 5** |  |  |
| Min |  |  |
| Median [IQR] | 0.10 [0.10, 0.10] | 0.10 [0.10, 0.10] |
| Mean (SD) | 0.13 (0.11) | 0.13 (0.05) |
| Max |  |  |
| Median [IQR] | 0.10 [0.10, 0.27] | 0.10 [0.10, 0.10] |
| Mean (SD) | 0.26 (0.32) | 0.22 (0.13) |
| **From Day 5 to Day 6** |  |  |
| Min |  |  |
| Median [IQR] | 0.10 [0.10, 0.10] | 0.10 [0.10, 0.10] |
| Mean (SD) | 0.11 (0.04) | 0.12 (0.04) |
| Max |  |  |
| Median [IQR] | 0.10 [0.10, 0.20] | 0.10 [0.10, 0.20] |
| Mean (SD) | 0.17 (0.11) | 0.18 (0.13) |
| **From Day 6 to Day 7** |  |  |
| Min |  |  |
| Median [IQR] | 0.10 [0.10, 0.10] | 0.10 [0.10, 0.10] |
| Mean (SD) | 0.10 (0.00) | 0.12 (0.04) |
| Max |  |  |
| Median [IQR] | 0.10 [0.10, 0.18] | 0.10 [0.10, 0.12] |
| Mean (SD) | 0.17 (0.12) | 0.18 (0.15) |

IQR: Interquartile Range; SD: Standard Deviation

| Characteristics | Dexmedetomidine group  (n=165) | Placebo group  (n=166) |
| --- | --- | --- |
| Sedatives, n (%) | 139 (84.2) | 142 (85.5) |
| Propofol | 139 (84.2) | 141 (84.9) |
| Ketamine | 57 (34.5) | 53 (31.9) |
| Etomidate | 37 (22.4) | 32 (19.3) |
| Sevoflurane | 37 (22.4) | 36 (21.7) |
| Opioids, n (%) | 153 (92.7) | 153 (92.2) |
| Morphine | 130 (78.8) | 131 (78.9) |
| Sufentanil | 102 (61.8) | 104 (62.7) |
| Oxycodone | 23 (13.9) | 20 (12.0) |
| Tramadol | 28 (17.0) | 31 (18.7) |
| Remifentanil | 15 (9.1) | 13 (7.8) |
| Codeine | 7 (4.2) | 6 (3.6) |
| Non-opioid analgesics, n (%) | 160 (97) | 157 (94.6) |
| Paracetamol | 159 (96.4) | 153 (92.2) |
| Nefopam | 77 (46.7) | 79 (47.6) |
| NSAID | 36 (21.8) | 34 (20.5) |
| Benzodiazepines, n (%) | 58 (35.2) | 59 (35.5) |
| Alprazolam | 32 (19.4) | 35 (21.1) |
| Midazolam | 24 (14.5) | 19 (11.4) |
| Oxazepam | 11 (6.7) | 9 (5.4) |
| Bromazepam | 1 (0.6) | 2 (1.2) |
| Loprazolam | 0 (0.0) | 1 (0.6) |
| Lorazepam | 2 (1.2) | 2 (1.2) |
| Clonazepam | 0 (0.0) | 1 (0.6) |
| Neuroleptics, n (%) | 12 (7.3) | 10 (6) |
| Haloperidol | 4 (2.4) | 5 (3) |
| Risperidone | 5 (3.0) | 4 (2.4) |
| Cyamemazine | 3 (1.8) | 1 (0.6) |
| Loxapine | 3 (1.8) | 1 (0.6) |
| Levomepromazine | 1 (0.6) | 0 (0.0) |
| Other, n (%) | 33 (20) | 47 (28.3) |
| Zopiclone | 27 (16.4) | 38 (22.9) |
| Hydroxyzine | 9 (5.5) | 11 (6.6) |
| Mianserine | 1 (0.6) | 2 (1.2) |
| Pregabaline | 2 (1.2) | 3 (1.8) |
| Melatonine | 1 (0.6) | 0 (0.0) |
| Corticosteroids | 2 (1.2) | 1 (0.6) |

**Table S2:** All concomitant treatments administered from inclusion to Day 7 in both groups.

NSAID: Non-Steroid Anti Inflammatory Drugs

**Table S3:** Main reason why CAM-ICU assessment and sleep quality evaluation were not performed or missing from Day 1 to Day 7.

|  | Dexmedetomidine group  (n=165) | Placebo group  (n=166) |
| --- | --- | --- |
| Day 1 |  |  |
| First evaluation (8 to 12 am), n (%) |  |  |
| Deep sedation (-4) | 4 (21) | 3 (18.8) |
| Unarousable (-5) | 12 (63.2) | 5 (31.3) |
| Under mechanical ventilation, n (%) | 37 (22.4) | 30 (18.1) |
| Second evaluation (4 to 8 pm), n (%) |  |  |
| Deep sedation (-4) | 2 (9.5) | 4 (21.1) |
| Unarousable (-5) | 8 (38.1) | 2 (10.5) |
| Hospital discharge | 0 (0) | 0 (0) |
| Day 2 |  |  |
| First evaluation (8 to 12 am), n (%) |  |  |
| Deep sedation (-4) | 2 (12.5) | 1 (8.3) |
| Unarousable (-5) | 7 (43.8) | 3 (25) |
| Under mechanical ventilation, n (%) | 11 (6.7) | 7 (4.2) |
| Second evaluation (4 to 8 pm), n (%) |  |  |
| Deep sedation (-4) | 3 (21.4) | 1 (9.1) |
| Unarousable (-5) | 4 (28.6) | 2 (18.2) |
| Hospital discharge | 1 (8.3) | 0 (0) |
| Day 3 |  |  |
| First evaluation (8 to 12 am), n (%) |  |  |
| Deep sedation (-4) | 1 (4.5) | 1 (5) |
| Unarousable (-5) | 4 (18.2) | 0 (0) |
| Under mechanical ventilation, n (%) | 8 (4.8) | 6 (3.6) |
| Second evaluation (4 to 8 pm), n (%) |  |  |
| Deep sedation (-4) | 2 (7.4) | 0 (0) |
| Unarousable (-5) | 3 (11.1) | 1 (4.5) |
| Hospital discharge | 1 (3.7) | 1 (4.5) |
| Day 4 |  |  |
| First evaluation (8 to 12 am), n (%) |  |  |
| Deep sedation (-4) | 4 (13.8) | 0 (0) |
| Unarousable (-5) | 2 (6.9) | 2 (8.7) |
| Under mechanical ventilation, n (%) | 7 (4.2) | 5 (3) |
| Second evaluation (4 to 8 pm), n (%) |  |  |
| Deep sedation (-4) | 3 (9.7) | 0 (0) |
| Unarousable (-5) | 2 (6.5) | 1 (3.7) |
| Hospital discharge | 1 (3.2) | 1 (3.7) |
| Day 5 |  |  |
| First evaluation (8 to 12 am), n (%) |  |  |
| Deep sedation (-4) | 2 (6.7) | 0 (0) |
| Unarousable (-5) | 2 (6.7) | 1 (3.3) |
| Under mechanical ventilation, n (%) | 7 (4.2) | 5 (3) |
| Second evaluation (4 to 8 pm), n (%) |  |  |
| Deep sedation (-4) | 3 (8.1) | 0 (0) |
| Unarousable (-5) | 1 (2.7) | 1 (2.9) |
| Hospital discharge | 1 (2.7) | 2 (5.7) |
| Day 6 |  |  |
| First evaluation (8 to 12 am), n (%) |  |  |
| Deep sedation (-4) | 2 (7.1) | 0 (0) |
| Unarousable (-5) | 1 (3.6) | 0 (0) |
| Under mechanical ventilation, n (%) | 7 (4.2) | 5 (3) |
| Second evaluation (4 to 8 pm), n (%) |  |  |
| Deep sedation (-4) | 1 (1.9) | 0 (0) |
| Unarousable (-5) | 1 (1.9) | 0 (0) |
| Hospital discharge | 7 (13.5) | 7 (14.6) |
| Day 7 |  |  |
| First evaluation (8 to 12 am), n (%) |  |  |
| Missing values | 48 (29.1) | 55 (33.1) |
| Deep sedation (-4) | 0 (0) | 1 (1.8) |
| Unarousable (-5) | 1 (2.1) | 0 (0) |
| Under mechanical ventilation, n (%) | 6 (3.6) | 2 (1.2) |
| Second evaluation (4 to 8 pm), n (%) |  |  |
| Deep sedation (-4) | 0 (0) | 0 (0) |
| Unarousable (-5) | 1 (1.4) | 0 (0) |
| Hospital discharge | 37 (52.9) | 29 (42) |

**Table S4:** Comparison of dexmedetomidine versus placebo on secondary outcomes: daily evaluation of sleep quality. Data are expressed as median and IQR.

| Outcomes | Dexmedetomidine group  (n=165) | Placebo group  (n=166) | P |
| --- | --- | --- | --- |
| LSEQ average score |  |  |  |
| Day 1  Missing | -5 (-13; 4.8)  57 | -10.5 (-19.5; 1.5)  59 | < 0.01 |
| Day 2  Missing | -6 (-15.6; 5)  41 | -9.5 (-18; 2)  39 | 0.049 |
| Day 3  Missing | -5 (-13; 2.5)  43 | -6 (-15; 4.5)  41 | 0.85 |
| Day 4  Missing | -4 (-14.5; 3)  42 | -2.5 (-10.4; 6)  42 | 0.31 |
| Day 5  Missing | -3.5 (-12; 6)  50 | -3 (-11; 7)  43 | 0.74 |
| Day 6  Missing | -0.5 (-10; 7)  51 | -1 (-10; 9)  48 | 0.99 |
| Day 7  Missing | -0.9 (-8.5; 13)  63 | 0 (-6; 9)  65 | 0.78 |
| Numerical scale (0-10) |  |  |  |
| Day 1  Missing | 5 (2; 8)  46 | 5 (2; 8)  47 | 0.91 |
| Day 2  Missing | 5 (4; 8)  47 | 5 (2; 8)  39 | 0.05 |
| Day 3  Missing | 5 (3; 7)  42 | 5 (3; 7)  48 | 0.48 |
| Day 4  Missing | 5 (3; 7)  52 | 6 (4; 7)  51 | 0.91 |
| Day 5  Missing | 5 (4; 7)  51 | 5 (4; 7)  53 | 0.89 |
| Day 6  Missing | 5 (4; 8)  53 | 6 (5; 8)  54 | 0.38 |
| Day 7  Missing | 6 (5; 8)  55 | 6 (4; 8)  67 | 0.56 |
| Sleep duration (hours) |  |  |  |
| Day 1  Missing | 4 (3; 7)  86 | 4 (2; 6)  81 | 0.52 |
| Day 2  Missing | 5 (3; 7)  79 | 4 (2; 6)  76 | 0.02 |
| Day 3  Missing | 5 (3; 6)  77 | 5 (3; 7)  70 | 0.44 |
| Day 4  Missing | 5 (3; 6)  77 | 5 (3.5; 7)  70 | 0.65 |
| Day 5  Missing | 5 (3; 7)  78 | 5 (4; 7)  76 | 0.59 |
| Day 6  Missing | 6 (4; 7)  74 | 6 (4; 7)  76 | 0.54 |
| Day 7  Missing | 5 (4; 7)  77 | 6 (3.5; 7.5)  86 | 0.25 |

IQR: Interquartile Range ; LSEQ: Leeds Sleep Evaluation Questionnaire ; Numerical scale is a numerical rating scale of sleep quality where 0 indicated worst possible sleep and 10 indicated best possible sleep

**Table S5:** Comparison of dexmedetomidine versus placebo on secondary outcomes: detailed sections of LSEQ during the 7 days of observation. Data are expressed as median and IQR.

| Outcomes | Dexmedetomidine group  (n=165) | Placebo group  (n=166) | P |
| --- | --- | --- | --- |
| Day 1 LSEQ |  |  |  |
| Getting to sleep  Missing | 2 (-4; 6.5)  48 | -1 (-6; 6)  47 | 0.06 |
| Quality of sleep  Missing | -3 (-5; 0)  48 | -4 (-6.5; 0)  48 | 0.07 |
| Awakening from sleep  Missing | -2.8 (-6; 0.9)  53 | -3.3 (-8.8; 0.6)  51 | 0.28 |
| Behaviour following wakefulness  Missing | -2 (-4; 0)  47 | -3 (-5; 0)  48 | 0.18 |
| Day 2 LSEQ |  |  |  |
| Getting to sleep  Missing | 1 (-4; 4)  38 | -2 (-6.4; 3)  34 | <0.01 |
| Quality of sleep  Missing | -3 (-5; 0.3)  38 | -4 (-7.5; -1)  34 | <0.01 |
| Awakening from sleep  Missing | -1.4 (-5; 2)  38 | -1.5 (-5; 2)  38 | 0.73 |
| Behaviour following wakefulness  Missing | -1.8 (-3.5; 0)  37 | -1 (-4; 0)  34 | 0.95 |
| Day 3 LSEQ |  |  |  |
| Getting to sleep  Missing | 0 (-5; 3)  40 | -0.1 (-5; 4)  34 | 0.50 |
| Quality of sleep  Missing | -3 (-5.8; 0)  37 | -3 (-7; 0)  35 | 0.31 |
| Awakening from sleep  Missing | -1 (-3.5; 2)  40 | 0 (-3; 3)  38 | 0.41 |
| Behaviour following wakefulness  Missing | -0.9 (-3.5; 1)  39 | -1 (-3.9; 1)  32 | 0.91 |
| Day 4 LSEQ |  |  |  |
| Getting to sleep  Missing | -1 (-5.8; 2)  41 | 0 (-4; 3)  37 | 0.19 |
| Quality of sleep  Missing | -2 (-5; 0)  40 | -2 (-5; 0.1)  38 | 0.87 |
| Awakening from sleep  Missing | 0 (-3; 3)  41 | 0 (-3; 4)  40 | 0.35 |
| Behaviour following wakefulness  Missing | 0 (-2; 2)  40 | 0 (-2.5; 2)  37 | 0.61 |
| Day 5 LSEQ |  |  |  |
| Getting to sleep  Missing | -1 (-5.2; 2.3)  45 | 0 (-4; 3.5)  39 | 0.10 |
| Quality of sleep  Missing | -1 (-5; 1)  45 | -2 (-6; 0.9)  40 | 0.29 |
| Awakening from sleep  Missing | 0 (-3; 3)  48 | 0 (-3; 4)  42 | 0.78 |
| Behaviour following wakefulness  Missing | 0 (-1.5; 2.3)  45 | 0 (-3; 2)  41 | 0.12 |
| Day 6 LSEQ |  |  |  |
| Getting to sleep  Missing | 0 (-4; 3)  47 | 0 (-4.5; 2.5)  45 | 0.80 |
| Quality of sleep  Missing | 0 (-4; 1)  46 | -1 (-5; 1.6)  45 | 0.37 |
| Awakening from sleep  Missing | 0 (-2.7; 3)  46 | 0 (-3; 5)  43 | 0.94 |
| Behaviour following wakefulness  Missing | 0 (-1; 3.5)  46 | 0 (-2; 3)  44 | 0.17 |
| Day 7 LSEQ |  |  |  |
| Getting to sleep  Missing | 0 (-3.8; 3)  57 | 0 (-4; 3.5)  64 | 0.99 |
| Quality of sleep  Missing | 0 (-4; 2.2)  59 | -1 (-4; 2.5)  64 | 0.64 |
| Awakening from sleep  Missing | 0 (-2.6; 6)  61 | 0.2 (-2; 5)  65 | 0.74 |
| Behaviour following wakefulness  Missing | 0 (-1.4; 5)  57 | 0 (-2; 3.5)  64 | 0.85 |

IQR: Interquartile Range ; LSEQ: Leeds Sleep Evaluation Questionnaire.

**Table S6:** Baseline creatinine level and daily renal component of the SOFA (Sequential Organ Failure Assessment) score.

|  | **Dexmedetomidine group** | **Placebo group** | **p** |
| --- | --- | --- | --- |
|  | **(n=165)** | **(n=166)** |  |
| **Baseline creatinine level (µmol/l), mean (SD)** | 84.9 (25.8) | 88.1 (30.7) | 0.32 |
| **Day 1, n (%)** |  |  | 0.7 |
| 0 | 127 (85.2) | 131 (86.2) |  |
| 1 | 13 (8.7) | 12 (7.9) |  |
| 2 | 7 (4.7) | 4 (2.6) |  |
| 3 | 1 (0.7) | 3 (2.0) |  |
| 4 | 1 (0.7) | 2 (1.3) |  |
| **Day 2, n (%)** |  |  | 0.67 |
| 0 | 120 (87.0) | 113 (84.3) |  |
| 1 | 8 (5.8) | 13 (9.7) |  |
| 2 | 8 (5.8) | 5 (3.7) |  |
| 3 | 1 (0.7) | 1 (0.7) |  |
| 4 | 1 (0.7) | 2 (1.5) |  |
| **Day 3, n (%)** |  |  | 0.61 |
| 0 | 86 (81.9) | 91 (83.5) |  |
| 1 | 14 (13.3) | 11 (10.1) |  |
| 2 | 2 (1.9) | 5 (4.6) |  |
| 3 | 1 (1.0) | 0 (0.0) |  |
| 4 | 2 (1.9) | 2 (1.8) |  |
| **Day 4, n (%)** |  |  | 0.98 |
| 0 | 77 (82.8) | 80 (83.3) |  |
| 1 | 12 (12.9) | 11 (11.5) |  |
| 2 | 2 (2.2) | 2 (2.1) |  |
| 3 | 1 (1.1) | 1 (1.0) |  |
| 4 | 1 (1.1) | 2 (2.1) |  |
| **Day 5, n (%)** |  |  | 0.77 |
| 0 | 73 (84.9) | 78 (87.6) |  |
| 1 | 9 (10.5) | 8 (9.0) |  |
| 2 | 2 (2.3) | 1 (1.1) |  |
| 3 | 1 (1.2) | 0 (0.0) |  |
| 4 | 1 (1.2) | 2 (2.2) |  |
| **Day 6, n (%)** |  |  | 0.5 |
| 0 | 57 (85.1) | 60 (80.0) |  |
| 1 | 6 (9.0) | 9 (12.0) |  |
| 2 | 4 (6.0) | 3 (4.0) |  |
| 3 | 0 (0.0) | 1 (1.3) |  |
| 4 | 0 (0.0) | 2 (2.7) |  |

SD: Standard Deviation

**Table S7:** Preplanned sub-group analysis for the primary outcome. Occurrence of PoD within the 7 days after surgery are expressed as number (%).

| Outcomes | Total | Dexmedetomidine group | Placebo group | P |
| --- | --- | --- | --- | --- |
| Modality of surgery |  |  |  |  |
| By-pass |  |  |  |  |
| Number of patients | 313 | 156 | 157 |  |
| PoD, n (%) | 39 (12.9) | 20 (13.3) | 19 (12.5) | 0.8 |
| Missing | 11 | 6 | 5 |  |
| Off-pump |  |  |  |  |
| Number of patients | 18 | 9 | 8 | 1 |
| PoD, n (%) | 1 (5.6) | 0 (0) | 1 (11.1) |  |
| Missing | 0 | 0 | 0 |  |
| Type of cardiac surgery |  |  |  |  |
| Valvular surgery |  |  |  |  |
| Number of patients | 120 | 55 | 65 |  |
| PoD, n (%) | 13 (11.4) | 3 (5.4) | 10 (16.1) | 0.08 |
| Missing | 6 | 3 | 3 |  |
| CABG |  |  |  |  |
| Number of patients | 128 | 68 | 60 | 0.71 |
| PoD, n (%) | 12 (9.7) | 7 (10.6) | 5 (8.6) |  |
| Missing | 4 | 2 | 2 |  |
| Combined surgery (valvular + CABG) |  |  |  |  |
| Number of patients | 53 | 25 | 28 |  |
| PoD, n (%) | 11 (21.2) | 7 (29.2) | 4 (14.3) | 0.19 |
| Missing | 1 | 1 | 0 |  |

CABG: Coronary Artery Bypass Grafting ; PoD: Postoperative Delirium.

**Appendix 1:** The cognitive failures questionnaire

******

**Appendix 2:** The PCL-5 standard form checklist

******

**Appendix 3:** The Leeds Sleep Evaluation Questionnaire (LSEQ). Each item is rated from -5 to +5. Negative score corresponded to negative effects on sleep quality.
